# Power to the people: Does Eterna signal the arrival of a new wave of crowd-sourced projects?

**DOI:** 10.1186/1471-2091-14-26

**Published:** 2013-10-23

**Authors:** Thomas A Rowles

**Affiliations:** 1 London, UK

## Introduction

What constitutes a scientist, and do you have to be a professional researcher to perform science?

These are questions that have unwittingly been asked in recent years by a number of crowd-sourcing initiatives, and none more so than one of the most recent: Eterna [1]. Following in the footsteps of Foldit [2], Eterna is an online puzzle game that is helping to forward our knowledge of how the primary sequence of RNA molecules determines their structure. Indeed, not only is Eterna doing a job that one would have thought would be restricted to highly-trained scientific researchers and complexly coded algorithms, but it also seems to be doing it better.

### How does Eterna work?

Before we proceed, we would encourage readers to go to the Eterna website, as this will provide a further understanding of the game than we are able to give here. However, to summarise: At the beginning of a typical Eterna round, players are presented with a string of adenine residues and a target conformation. They are then challenged to form the string into the target simply by changing adenines to other nucleotides and thereby taking advantage of the interactions between them. It sounds simple, but it’s not long before you have to start taking account of how the placement of a particular nucleotide will affect its neighbour, as well as the effects of the orientation of each pair. In preparing this article we have only played through some of the simpler levels of the game, but even there the sense of achievement at coaxing a string into the target shape is palpable, and at this level the game hasn’t even played its trump card. Achieve 10,000 points and you earn the right to submit sequences to be synthesised in the laboratory. The developers then feedback on the accuracy of solutions. Further, at 20,000 points the players earn the right to design their own puzzles.

Why would the developers go to the trouble of synthesising the designs of players though? Surely the game, though an entertaining diversion, doesn’t warrant this? Well, as alluded to above, game players are discovering previously unknown rules about how the primary sequence of RNA dictates its structure. In fact, players quickly began to outperform algorithms that had been specially designed by experts for the task, and the rules that they have discovered are now being incorporated into the next generation of algorithms. Does this mean that the term 'player’ is no longer appropriate for those who have attained this level? Should we really be starting to refer to them as scientists? In order to investigate this, we were very interested in talking to some of the players to explore their motivation, and what they thought the future of this type of this research might be. We were kindly provided with the email addresses of several of the top game players by the developers of Eterna, and sent them a number of questions related to their experience with the game. An exploration of the responses received is presented below.

### What makes an Eterna player?

To generalise, it would appear that there are two broad types of player, though most will obviously sit somewhere between these two extremes. These two types can be neatly summed up by the response of one player when asked what makes a good Eterna player:

“There are different profiles of players in Eterna. Some may be only concerned by their ranking, and then, only their ability to solve puzzles will count towards defining how good they are. Then, there are those like me who are in this for the science and the research.” (Nando)

In terms of attributes shared by Eterna enthusiasts, while agreeing that there is a broad spectrum of types of players, the following two responders summed up a number of useful qualities:

“There are all kinds of players, but most of the top 10–20 players have 1) an analytical mind, 2) a methodical approach to problem solving, 3) patience to work through puzzles that take a lot of time to solve, 4) lots of time on their hands. (Robert Jensen)

“I think anybody can be a good Eterna player, but it helps to be competitive, curious and have an addictive personality.” (Kevin Cabral)

If we, rather unscientifically, assume that these two responses accurately describe the personalities of typical high-level game players, then I think it would be difficult to argue that there are not marked similarities between the players and a significant number of research scientists.

### Discovering Eterna

The first encounter with Eterna for many players seems to have come through the popular media, with both a Wired magazine article and the PBS television programme Nova scienceNOW receiving a number of mentions, as did various online science blogs, perhaps belying an underlying general interest in science. However, what seemed to draw many of the players in was the simple appeal of puzzle solving. The lure of RNA creation and scientific discovery seems to take hold later.

“I like solving RNA puzzles, but I would be almost as happy playing Bejeweled. What makes me come back is the lab and the lab results in particular.” (Eli Fisker)

“I have always loved to work puzzles and got hooked on these. When I tried the first few puzzles I couldn’t solve any of them. They made no sense to me so I was both annoyed and challenged. I slowly figured out how to solve most of them although there are still a couple of puzzle types that I can’t solve. So I keep coming back and I will get them in the end!” (Aldwyn Hyatt)

“The initial appeal was the quick 'success’ of solving an RNA design puzzle. Later it was the challenge of designing RNA to be folded and tested in vitro.” (Jeff Anderson-Lee)

One of the things that became clear as we read through the responses is that while none of the players had any higher-level scientific education, the majority did express an interest in scientific research, whether this be through the popular media or, in one case, through the reading of scientific papers and their dissemination to other players. Despite this, it was generally felt that a scientific background did not constitute an advantage when playing Eterna. Indeed, as one respondent pointed out, experienced non-scientific players generally beat researchers who are new to the game, but may have studied the intricacies of molecular biology for years:

“I think that the ability to see patterns, and come up with strategies, helps to make a good player. The fact that non-scientifically trained, but somewhat experienced players, can beat PhD micro-biology students who have not had much practice with the game in designing in vitro RNA sequences, shows that lack of formal training is definitely not a barrier to entry, and formal training is not a guarantee of success.” (Jeff Anderson-Lee)

One of the players interviewed also put forward a quote, made earlier this year by a different player, that attempted to provide some explanation of why this might be:

“The difference between us and scientists (and there are scientists among us), is we have no idea what should work so we try everything. We’re more creative.” (WaterontheMoon)

### Is there a role for Eterna in research?

Moving away from the game players specifically, we were obviously intrigued by the Eterna initiative itself and what the future could hold for it. While it is clearly already playing a role in research, as it furthers our knowledge of how RNA folds, we wanted to know whether the players thought that crowd-sourcing might be the road down which science will travel in the coming years. Interestingly, despite the obvious respect and praise that they have for the initiative, the players interviewed were refreshingly pragmatic about its place in the wider world of scientific endeavour:

“I think crowd-sourced experiments, like Eterna, will be a good supplement to what science is already being carried out.” (Eli Fisker)

“…there is a class of problems in science, requiring to deal with extremely large amounts of data and/or with extremely complex algorithms (those called 'NP-hard’ by computer scientists), and I do believe that a crowd can prove very useful and powerful in those cases.” (Nando)

“Crowd-sourcing has its place in scientific research, but will not, nor should not, replace the individual, the 'drop a flask, pick it up, make discovery’ type of research going on in labs around the world.” (Anonymous)

### Does Eterna have the potential to become an educational tool?

So with an important but perhaps limited role for crowd-sourcing envisaged for research, what did the interviewees think of the use of Eterna in an educational capacity? There is some indication that school groups had been on 'trips’ into the game, so it appears that this might already be happening.

“We have had teachers bring their classes into the game. I think it is a good way to learn, not just about RNA, but also about doing science.” (Eli Fisker)

“I’m pretty sure it is a fun way to present RNA to high-schoolers. And there’s already been a few 'visiting classes’ in the past.” (Nando)

In general though, up to this point there did not seem to have been much consideration that the game could be used in a formal education capacity such as we had envisaged, though some players did see how there was potential in this.

“I think that as soon as your homework assignment is to perform experiments with RNA by playing a video game online, students are not only going to be enthusiastic to learn about science, they will also learn more and perhaps get better grades. Some of these students may even be inspired to become scientists.” (Kevin Cabral)

What struck us, however, and what we had not envisaged, was just how much the game had taught its players, and how much their interest in the underlying concepts had been piqued.

“Is it a useful teaching tool? Well, ten months ago, I didn’t know the first thing about nucleic acids (other than they existed), and if one takes a look at the materials I uploaded on the wiki, I think it could be said that I’ve been taught quite a few things. Yet, all of it was my own seeking for more information…Since I started playing and learning about RNA, there hasn’t been a day where I haven’t learned something new about the RNA world.” (Nando)

### Interactions between players and community building

Even more excitingly than what players had learned about RNA from the game, we had not envisaged just how much education and instruction would be going on between the players.

“…playing Eterna has taught me a little about RNA just from working puzzles, and many players in the community openly share and teach in-game what they know and what they have learned about folding RNA.” (Lee Bickle)

“Personally, I maintain on my profile page many links to papers that the more science orientated may be interested in reading. Like the other players, I post informative pictures to promote better understanding within the game, especially for newbies but also for advanced players to consider.” (Chris Couteau)

What if player teaching and education weren’t the only un-envisaged factors to come out of Eterna though? What if players began to bring their own unique skills to the party and to start to alter and develop the very nature of the game? Fantastically, this is already starting to happen.

*“My greatest achievement is most likely the recent help in getting the scripting interface discussion really moving and bringing software tools sets to their attention to aid the porting of software to JavaScript. As a result some of the Vienna RNA software suite has been ported to JavaScript…I have helped establish contacts with the VARNA*[3]*team to facilitate porting their graphic display software from Java to JavaScript as well, though the follow through has yet to occur as much.” (Chris Couteau)*

*“I could say that I have some reasons to feel relatively proud of having written a software capable of playing and competing with human players in this game (the bot is currently ranked 30*^*th*^*).” (Nando)*

There is an argument that the term 'player’ no longer fits these individuals, but that 'scientist’ isn’t quite right either. It seems as if they are starting to occupy a grey area somewhere between player and developer. There is a level of creativity and drive here that Eterna seems to have drawn out, and which is contributing to the further development of the game in a way that we imagine the developers could never have predicted. So how do the developers feel about this. Is there a concern that the players are overstepping certain boundaries and tinkering with things that they shouldn’t be playing with? On the contrary; it appears that this is a development that designers actively welcome. Indeed, they hold biweekly development meetings with players to discuss thoughts, ideas and updates to the game.

One of the strongest indications to come out of the development work described above, and the obvious instruction and education that goes on between players, is the strong sense of community that exists between the top game players, and how much of the appeal of playing it is social, which will of course foster collaboration and creativity, particularly in an environment that appears to be entirely non-judgemental and cooperative.

“Most of my success in the game is owed to other players who have spent much time and effort analyzing, compiling, & documenting what they’ve learned and engaging in dialog with me about the game.” (Lee Bickle)

## Conclusions

To return to our original question, what constitutes a scientist, and are the Eterna players challenging this concept? It could certainly be argued that the players possess many of the attributes that define research scientists. They are adept at recognising patterns and display formidable problem solving skills. Perhaps even more importantly, they also display a devotion to what they do that sometimes seems to border on the obsessive. However, perhaps what we’ve really learned in speaking to the players is just how reductive our original question was. In fact, maybe this is where the true innovation of Eterna lies: In realising that the traditional reductionist approach, where we would think that the only people qualified to undertake research are conventionally trained scientists, is not the only way to go about things, and in having the foresight to design the tools to take advantage of a previously underutilised workforce. In the end, it doesn’t really matter what attributes Eterna players have, and whether we should consider them as scientists or not (for the record, the term favoured by the developer Dr Rhiju Das, and which seems to have been adopted by the scientific community in general, is 'citizen scientists’). The simple fact is that these are intelligent and dedicated people who have been given the opportunity to do something meaningful and worthwhile, and at the same time challenging and fun, and who have grasped this opportunity with both hands.

It could be argued that the word 'foresight’ above is misused. After all, the original aim of Eterna was to utilise the so called 'wisdom of crowds’, and it could not have been envisaged at inception that it would select for a group of individuals who would have the skills and the inclination to become involved in the future development of the game. However, the chance was taken, and the opportunities were given to allow the game to develop and evolve in this organic way. In essence, there was a drive and an enthusiasm for exploring the unknown potentials, which perhaps we are tending to see less and less in modern science. The game does not seem to have been developed with a view to the next big publication, nor is granting extra development power to the players going to assist in this. Moreover, it would appear to have been a simple exploration, both of the potential of the tools and of the underlying science of RNA folding, driven by a passion and a joy in what was being achieved. Interestingly, for many of the players, these seem to be the self-same reasons that they persist in putting so much time and effort into both playing the game, and forwarding its development. Furthermore, this spirit appears to have taken hold with the developers, as they are now enabling other laboratories to use Eterna for their own crowd-sourced RNA design and modelling projects. In the words of Dr Das: “We have been democratizing science for gamers, but now we're democratizing the creation of games for any scientist – expert or citizen!”

Is the future of science going to be based around crowd-sourced experiments? Our opinion, and that of Eterna players, is no. However, it is a new, potentially powerful tool in our arsenal that should be considered for 'big-data’ projects. *BMC Biochemistry* is very excited about seeing what new directions these initiatives take us, and we hope that we will be considering more and more manuscripts that integrate results obtained through citizen science initiatives with more 'traditional’ laboratory results in the future.

In addition, what we should also take from the Eterna project is that we should be more creative about developing solutions to scientific problems, and that putting the right tools into the hands of the right people, even if they are not the ones that you might have been expecting, can yield incredible results. We like to think that, thanks to the open-access nature of *BMC Biochemistry*, important science will not only be performed by members of the public, but that the results of their efforts will also be freely available for them to appreciate.
